# Falls among elderly: evidence from hospital settings in Georgia

**DOI:** 10.1093/eurpub/ckac131.419

**Published:** 2022-10-25

**Authors:** N Pitskhelauri, T Dochviri, K Akhobadze, N Chkhaberidze, N Chikhladze, M Kereselidze, C Peek-Asa, M Adina Coman

**Affiliations:** 1 TSU, Tbilisi, Georgia; 2 National Center of Disease Control and Public Health, Tbilisi, Georgia; 3 University of California, San Diego, USA; 4 Babes-Bolyai University, Cluj-Napoca, Romania

## Abstract

**Introduction:**

Falls are one of the major public health problems and a leading cause of injury-related morbidity and mortality globally. Around of 80% of fall deaths occur in low- and middle- income countries. Falls are the most common cause of hospitalization among the population aged 65 years and older. According the data from Global Burden of Disease (GBD) Study in Georgia, DALYs from falls exceed the average in LMICs. The aim of this observational study was to explore epidemiological characteristics of fall-related injury among hospitalized elderly patients in Georgia in 2020.

**Methods:**

This study was designed in the framework of the project iCREATE: Increasing Capacity for Research in Eastern Europe’ funded by the NIH (2D43TW007261). All patients aged 65 and older admitted to hospital settings due fall-related injury in 2020 were identified from the Hospital Registry (included a total of 152 hospitals in the country) of the National Center for Disease Control and Public Health of Georgia.

**Results:**

A total of 7159 injured patients age 65 and above were admitted to hospitals in Georgia, and among them 4213 were hospitalized due to the falls (60%). Males comprised 30,7% of cases and females - 69,3%. The most common source of hospital arrival was ambulance 2791 (65,0%), followed by private/public transport 1432 (33,3%). The highest number of injuries (43,5%) occurred in Tbilisi (capital of Georgia). The leading cause in fall-related hospitalization was fall on same level. Pelvis and the head were the most common body regions injured accounting for 48,0% and 22,9% of cases, respectively. The highest LOS was 2014 (SD 6,161). 111 patients died due to falls related injury during the study period.

**Conclusions:**

This study indicates that fall- related injury prevention among elderly population should become a key priority area in Georgia. There is a need for elaborating relevant preventive interventions of falls injuries in population aged 65 years and older.

**Key messages:**

• Falls are the leading cause of hospitalization of patients 65 years of age and older.

• Fall risk awareness campaigns should be the first step to fall prevention among elderly.

